# The resistome of commensal *Escherichia coli* isolated from broiler carcasses “produced without the use of antibiotics”^a^

**DOI:** 10.1016/j.psj.2022.101770

**Published:** 2022-02-01

**Authors:** Lucia Gambi, Cecilia Crippa, Alex Lucchi, Alessandra De Cesare, Antonio Parisi, Gerardo Manfreda, Frédérique Pasquali

**Affiliations:** ⁎ Department of Agricultural and Food Sciences, Alma Mater Studiorum – University of Bologna, 40127, Italy; † Department of Veterinary Medical Sciences, Alma Mater Studiorum – University of Bologna, 40064, Italy; ‡ Istituto Zooprofilattico Sperimentale della Puglia e Basilicata, Putignano (Bari), 70017, Italy

**Keywords:** antimicrobial resistance genes, antibiotic-free, commensal Escherichia coli, broiler carcasses

## Abstract

Several strategies have been in place in food animal production to reduce the unnecessary use of antimicrobial agents. Beyond the monitoring of their use, the evaluation of the effect of these strategies on the occurrence and types of antimicrobial resistance (**AMR**) associated genes is crucial to untangle the potential emergence and spread of AMR to humans through the food chain. In the present study, the occurrence of these genes was evaluated in commensal *Escherichia coli* isolated from broiler carcasses “produced without the use of antibiotics” in 3 antibiotic-free (**AB**-free) farms in Italy in 2019. Sequenced data were analyzed along with publicly available genomes of *E. coli* collected in Italy from the broiler food chain from previous years (2017–2018). The genetic relationships among all 93 genomes were assessed on *de novo* assemblies by *in silico* MLST and SNP calling. Moreover, the resistomes of all genomes were investigated. According to SNP calling, genomes were gathered in three clades. Clade A encompassed, among others, ST117, ST8070, and ST1011 genomes. ST10 belonged to clade B, whereas Clade C included ST58, ST297, ST1101, and ST23 among others. Regarding the occurrence of AMR genes, a statistically significant lower occurrence of these genes in the genomes of this study in comparison to the public genomes was observed considering the whole group of genes as well as genes specifically conferring resistance to aminoglycosides, β-lactams, phenicols, trimethoprim, and lincosamides. Moreover, significant reductions were observed by comparing the whole group of AMR associated mutations, as well as those specifically for fluoroquinolones and fosfomycin resistance. Although the identification of 3° generation cephalosporin resistance associated genes in AB-free *E. coli* is a concern, this study provides a first indication of the impact of a more prudent use of antimicrobial agents on the occurrence of AMR genes in Italian broiler production chain. More studies are needed in next years on a higher number of genomes to confirm this preliminary observation.

## INTRODUCTION

The inappropriate use of antibiotics has been spotted as one of the drivers of antimicrobial resistance. In the animal production area, the widespread use of antimicrobials has been due to their use not only as therapeutic agents, but also as growth promoters, metaphylactic, and prophylactic agents worldwide. This overuse increased especially in the second half of the 19th century and boosted the emergence of AMR bacteria and their AMR genes which can spread and be transmitted to humans through the food chain.

In 2017, the total world consumption of antibiotics intended for veterinary use was around 85,330 tons (OIE, 2021). Out of 160 countries in the world, 26% were still using antibiotics as growth promoters in 2019 (OIE, 2021). In Europe, despite the ban of antibiotics as growth promoters and a reduction of sales of 34.6% in 25 of the 31 countries reporting data from 2011 to 2018 in food producing animals, the sales were higher than 100 mg/PCU in 9 countries in 2018 (EMA, 2020).

One of the animal food productions under the lens has been broiler production due to the high market value, volumes of production and the restraints linked to individual treatment of sick animals, which forces veterinarians to treat the whole flock as a metaphylactic approach. In this view, a great effort has been put in place in the poultry sector to reduce the burden of AMR. In Italy, where the majority of poultry production belongs to big integrated companies, a holistic approach has been followed with intervention strategies aiming at improving the quality of chicks, feed and water as well as transport, biosecurity, welfare, precision farming and education. These improvements drove the way to the implementation of antibiotic free farms from which the produced meat is labeled as “reared without the use of antibiotics”. In these farms, in case of bacterial disease evidence, the whole flock is treated with antibiotics and the label is removed from the produced meat. Thanks to the high level of integration and the efforts made by the poultry industry, this approach in the Italian poultry sector was effective in reducing up to 82% the sales of antibiotics in 2018 in comparison to 2011 (UNAITALIA, 2019). Beyond this promising result, Italy has been recognized as a case study of good practices for reducing antimicrobial use in poultry farming. The EIP-AGRI focus group reported education and knowledge transfer as the most important action to further promote the reduction of antimicrobial use in poultry farming in the future and cited Italy as a good example for organizing training activities for veterinarians, farmers, and other stakeholders. Moreover, Italy has been reported as case study of good practices for the certification of private standards for meat and egg production “reared without the use of antibiotics” as well as for the attention to animal welfare through the request of a big retailer to its suppliers to adhere to the welfare standards of the CReNBA (Italian National Reference Centre of animal welfare) (EIP-AGRI Focus Group, 2021).

Besides the monitoring of antimicrobial use, it is of paramount importance to evaluate the effective impact of implemented strategies on the antimicrobial susceptibility of AMR bacteria. *Escherichia coli* is considered a good indicator of the selective pressure exerted using antimicrobials on intestinal populations of bacteria in food animals. For this reason, a harmonized monitoring of phenotypic resistance to several antimicrobial classes in commensal indicator *E. coli* from food producing animals is in place since more than a decade now (EFSA and ECDC, 2021). Recently, for the first time in Italy and exclusively for poultry, a decreasing trend was observed in phenotypic resistance to ciprofloxacin, amoxicillin and tetracycline in commensal indicator *E. coli* isolated from broilers in the period 2014–2018 (EFSA and ECDC, 2021). Besides indicator *E. coli* isolated from the gut of broilers, commensal *E. coli* isolated from broiler carcasses represent a good target not only for the evaluation of AMR bacteria in animals but also for their potential transmission to humans compromising the success of antimicrobial treatments. Unfortunately, along with the huge amount of data on phenotypic resistance linked to monitoring activities at national and international level, few data are systematically collected on the genotypes of AMR bacteria. Data on the occurrence of antimicrobial resistance associated genes are crucial to untangle the potential emergence and spread of AMR to humans through the food chain.

This study aimed at evaluating the occurrence of antimicrobial resistance associated genes of commensal *Escherichia coli* isolated from broiler carcasses “produced without the use of antibiotics” in antibiotic-free farms in Italy in 2019. Sequence data were compared to publicly available genomes of *E. coli* collected in Italy from the broiler food chain from previous years (2017–2018).

## MATERIALS AND METHODS

### Sample Collection

Forty-five carcasses of Ross 308 broilers were collected at slaughterhouse. These carcasses belonged to birds reared in 3 intensive antibiotic free (AB-free) poultry farms in Italy. The 3 rearing cycles were held in October-November 2019. In particular 15 carcasses per farm were collected. Animals were slaughtered at 33, 35, and 34 days of age. Tracks of the same kind were used for animal transportation. The travel between each farm and the slaughterhouse was similar for all tested groups and lasted within 1 h.

### Isolation and Identification of *Escherichia Coli*

*E. coli* was isolated from 45 broiler carcasses. Briefly 5 g of neck skin was added to 45 ml of Buffer Peptone water (BPW, ThermoFischer, Milan, Italy). After incubation at 37 ± 1°C for 24 h, a loopful from BPW was streaked on MacConkey agar (ThermoFischer). Plates were incubated at 37 ± 1°C for 24 h. Five colonies per plate were submitted to biotyping.

Biotyping was performed using RapID ONE System (ThermoFisher Scientific). In case of confirmed *E. coli*, only one isolate per sampled carcass was retained for downstream analyses.

### Whole Genome Sequencing and ***de novo*** Assembly

Genomic DNA was extracted and purified using MagAttract HMW DNA Kit (Qiagen, Hilden, Germany). Libraries were built using the Nextera XT DNA Library Preparation Kit (Illumina, Milan, Italy) and sequenced on Illumina MiSeq Platform which generates tagged 250 bp paired end reads. Unless otherwise indicated, all subsequent genomic analyses were performed on Galaxy Trakr platform (Gangiredla et al., 2021). After performing a quality cleaning through TRIMMOMATIC, reads were *de novo* assembled using Shovill v.1.0.4, a standardized and fully automatic open-source pipeline for bacterial genome assembly. The quality assessment of the assemblies was verified using QUAST.

Sequencing data are available at NCBI Database under BioProject accession number PRJNA760335.

### Multi-Locus Sequence Typing

For the assignment of genomes to ST-types, *de novo* assemblies were submitted to Mlst v2.19.0, an open-source software for *in silico* MLST (Maiden et al., 1998).

### In Silico Serotyping

Serotypes of genomes were determined using the pipeline EURL VTEC WGS PT v3.0 available at Galaxy Trakr (Gangiredla et al., 2021).

### In Silico Phylo-typing

For the assignment of genomes to A, B1, B2, C, D, E, F and G phylogroups *de novo* assemblies were submitted to ClermonTyping (Beghain et al., 2018; Clermont et al., 2019).

### SNP Calling

SNP calling was performed using the open-source software snippy v4.5.0 applied on assemblies. Snippy is a rapid haploid variant calling and core genome alignment open-source tool. The pipeline includes several tools that align reads or assemblies from each isolate to a reference genome and then identifies variants among the alignments. The *E. coli* str. K-12 substr. MG1655 assembled genome (NCBI accession N° GCA_000005845.2 ASM584v2) was used as an internal reference for SNP calling. Based on the core SNP alignment, a high-resolution phylogeny tree was built including the conserved nucleotide variant sites shared by all genomes. PhyML v3.3.2 was used to analyze the SNP differences between isolates based on maximum likelihood algorithm and phylogenetic trees were visualized with iTOL (Letunic and Bork, 2021). Finally, a pairwise SNP distance matrix was built using snp-dists v0.6.3.

### Resistome

Analyses of the resistome of all genomes were performed using ABRicate v.0.7. This tool performs a BLAST search of genes using ResFinder database. Genomes were considered positive for AMR gene when the gene showed ≥60% of coverage and ≥98% of identity. Point mutations associated with antimicrobial resistance were identified through PointFinder in Center for Genomic Epidemiology website (CGE - https://cge.cbs.dtu.dk/services/ResFinder/, accessed on 24 of April 2021).

### Comparison with Public Genomes

Public genome data were downloaded the 19/04/2021 from NCBI pathogen detection web site (www.ncbi.nlm.nih.gov/pathogens/) for comparison. The key words and filters we used for search were: - Location: Italy. - Isolation source “broiler meat” “broiler” “chicken manure” “chicken breast” “chicken farm” “chicken” “chicken meat” “chicken carcass” “chicken meat raw” “chicken wings” “chicken trachea” “retail chicken” “chicken wing” “chicken thigh” “chicken hamburger”, Isolation type: “environmental/other”- Collection date: from 2016-01-01 to 2021-05-04. In total 54 public *E. coli* genomes isolated from broiler feces and broiler meat in Italy in 2017–2018 were retrieved (NCBI BioProjects PRJNA528851 and PRJNA602821). Upon request, authors of submission PRJNA602821 certified the origin of samples to be broilers reared in conventional systems. In order to compare presence/absence of AMR genes on the genomes of the present study in comparison to public genomes, statistical analysis was performed applying the chi-square test by the Excel implemented function CHISQ.TEST, *P* value < 0.05 was considered significant.

## RESULTS AND DISCUSSION

### Isolation of *E. Coli*

Thirty-nine isolates of *E. coli* were confirmed by biotyping and retained for downstream analyses: 12 from farm A, 12 from farm B and 13 from farm C.

### Whole Genome Sequencing and de Novo Assembly

After de *novo* assembly, the generated draft whole genome sizes of newly sequenced genomes ranged from 4,719,736 bp to 5,641,978 bp. The number of contigs ranged from 76 to 495 and N50 from 31,498 to 25,4657 bp.

### In Silico MLST-typing, Serotyping and Phylo-typing

MLST and *in silico* serotyping results revealed a high genetic diversity among the 93 *E. coli* genomes analyzed. The MLST and *in silico* serotyping gathered genomes in 39 already described ST-types and 60 serogroups respectively. The most frequently identified ST-types in newly sequenced *E. coli* were ST10 (6 isolates) and ST8070 (5 isolates). By *in silico* serotyping ST10 genomes were further separated in 4 serogroups: O5:H12, O176:H32, O132:H32, O99:H33. All ST8070 genomes belonged to O112ab:H20. ST10 was previously found in humans and food samples and considered ubiquitarian (Matamoros et al. 2017). Three newly sequenced *E. coli* genomes belonged to ST4642 and O116:H8. Interestingly no scientific publications were found on this ST-type indicating it is probably rare. Four samples belonged to ST58 (2 belonging to O?:H37 and 2 to O8:H25), 3 to ST1011 (2 O166:H45 and 1 O160:H12) and 3 to ST1101 (all three O39:H7). In particular ST58 and ST 1011 were previously described as ubiquitarian (Elnahriry et al., 2016; McKinnon et al., 2018) These ST-types are rarely associated with serious diseases in animals and humans. Other identified ST-types were ST162 (2), ST297 (3), ST4512 (1), ST117 (1), ST1324 (1), ST1251 (1), ST665 (1), ST3107 (1), ST2040 (1), ST1276(1), ST57 (1), ST6446 (1). Among these ST-types, ST117 is of particular interest. The newly sequenced ST117 genome belonged to O188:H4 and phylogroup G previously described as encompassing virulent and antimicrobial resistant strains (Clermont et al., 2019).

The most frequently identified ST-types in public genomes were: ST295 (5), ST117 (5) and ST9862 (4) in genomes of BioProject PRJNA602821 and ST117 (5) and ST23 (3) in genomes of BioProject PRJNA528851. Both ST-types, ST23 and ST117, were previously described as belonging to EXPEC strains suggesting them as potential pathogens (Manges et al., 2019). In particular, all ST 23 public genomes belonged to serogroup O78:H4 and phylogroup A, already described in the literature as gathering APEC strains (Dziva et al., 2013). ST117 public genomes belonged to different O serogroups but all were H4 and phylogroup D (5 genomes, PRJNA528851) or G (5 genomes, PRJNA602821). Both phylogroups D and G were previously described as gathering EXPEC strains (Manges, 2016; Clermont et al., 2019). Interestingly ST9862 differs from ST23 exclusively for *purA* locus (allele 13 in ST23 and allele 739 in ST9862). ST9862 genomes belonged to O78 serogroup (specifically O78:H9) as ST23 genomes but differently from ST23 they clustered in phylogroup C. Further in-depth analyses are required to investigate the potential pathogenicity of these ST23, ST117, and ST9862 public isolates.

### SNP Calling

**Figure 1:**
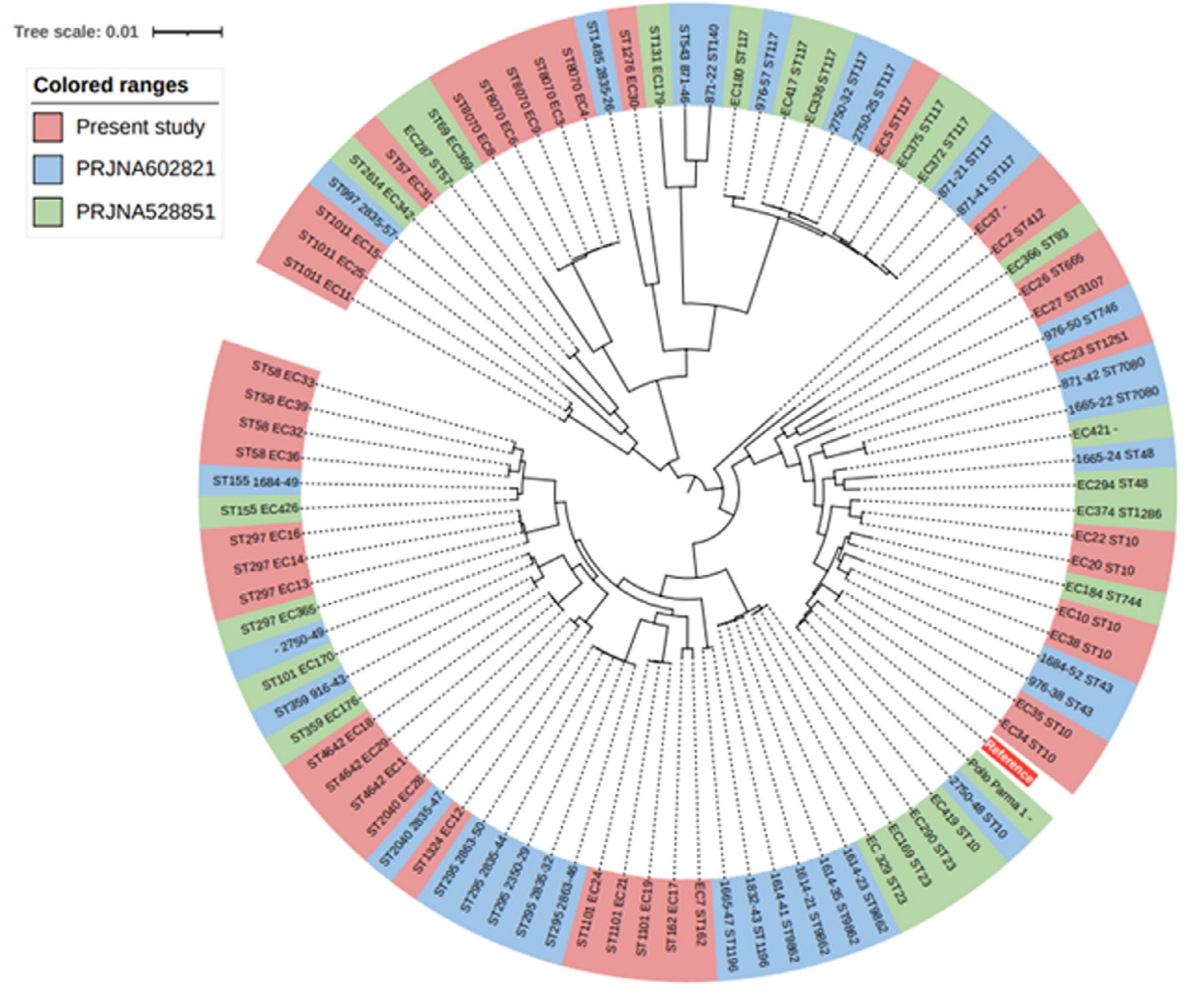
Maximum likelihood phylogenetic tree of Italian *E. coli* genomes from the poultry chain.

The maximum likelihood phylogenetic tree gathered Italian *E. coli* genomes from the poultry chain in three clades (Figure 1). Clade A was primarily including ST117, ST8070, and ST1011 genomes independently from the BioProject. ST10 belonged to clade B, whereas Clade C included ST58, ST297, ST1101, and ST23 among others (Figure 1). Regarding newly sequenced genomes, pairwise distances ranged from 1 to 46570 SNPs confirming the high genetic diversity shown by MLST. Additionally, SNPs calling was able to further differentiate isolates belonging to the same ST-type. Few *E. coli* genomes showed high pairwise similarity with differences from 1 to 46 SNPs. As expected, higher similarity was observed within ST-types and within farms (EC13, EC14, EC16 of farm B - ST297; EC15 and EC25 as well as EC21 and EC24 of farm B – ST1011; EC20 and EC22 of farm A – ST10; EC32 and EC36 as well as EC33 and EC39 of farm C – ST48). Interestingly genomes EC1 of farm A, EC18 of farm B and EC29 of farm C (all 3 belonging to ST4642 and O116:H8) were also closely related. Further analyses should be performed to confirm the potential epidemiological link within these three isolates.

### Resistome of Commensal E. Coli Collected From the Italian Broiler Food Chain

Based on WGS analysis, the occurrence of AMR associated genes was studied in the newly sequenced *E. coli* genomes from broiler carcasses reared in 2019 without the use of antimicrobial agents in Italy. In parallel the occurrence of the same AMR associated genes was evaluated in a comparable number of publicly available *E. coli* genomes isolated in Italy from the broiler food chain from 2017 to 2018. Ten newly sequenced *E. coli* genomes (26%) and none of the public *E. coli* genomes were completely lacking AMR genes. Seventeen newly sequenced *E. coli* genomes (44%) and 50 public *E. coli* genomes (93%) showed 3 or more AMR genes belonging to different antimicrobial classes. No correlations were found between types or number of AMR genes and ST-types. This is in agreement with the frequent localization of AMR genes in the accessory part of the genome which can be horizontally transferred between bacteria of different ST-types and/or species. On the other end, MLST is based on the genetic sequence of species-specific genes located in the core part of the genome (Maiden et al., 1998).

A total of 43 different AMR genes and 13 different AMR mutations were detected (Table 1). In the newly sequenced group, a total of 111 AMR genes were found in the 39 *E. coli* genomes analyzed, with an occurrence of AMR genes of 5.81% (Table 1). The *tet*(A) gene (38.5%), the *bla*TEM-1 (33%), the *strB* gene (30.8%), the *aadA1* gene (30.8%) and the *sul2* gene (28.2%) were the most frequently detected (Table 1). In the group of 54 public genomes, a total of 368 AMR genes were found, with an occurrence of 14.8%. The *bla*TEM-1 (77.8%), the *mdfA* gene (53.7%), the *tet(A)* gene (50%) and the *sul2* gene (40.7%) were the most frequently detected. Regarding point mutations, 13 AMR associated SNPs were identified in the newly sequenced group with an occurrence of 2%. In comparison, 82 AMR SNPs were found in the public genomes with an occurrence of 8.9% (Table 1).

**Table 1:** Resistome of genomes of this study and public genomes of *E. coli* isolated from the Italian broiler production chain.

Antimicrobial class	Type of genes or mutations	N (%) of genes in		*P* value
		Genomes of this study	Public genomes	
*Antimicrobial resistance associated genes*				
Aminoglycosides	aac(3)-Iid, aac(3)- ant(3′')-Ia, aadA1,aadA2, aadA5, aadA13, aph(3′')-Ib, aph(3′')-Ia, aph(6)-I, strA	37 (6.78%)	84 (11.11%)	0.00761
B-lactams	blaCTX-M-15, blaEC, blaTEM-1, blaTEM-1C, blaTEM-104, blaTEM-106, blaTEM-135	21 (5.98%)	69 (14.20%)	0.00012
Phenicols	catA1, cmlA1, floR	2 (1.71%)	13 (8.02%)	0.02101
Trimethoprim	dfrA1, dfrA5, dfrA12, dfrA14, dfrA17	8 (4.10%)	30 (11.11%)	0.00648
Lincosamides	lnu(F), lnu(G)	0 (0%)	14 (12.96%)	0.00094
Macrolides	mef(B), mph(A), mph(B)	1 (0.85%)	5 (3.09%)	0.20478
Fluoroquinolones	qnrB5, qnrB19, qnrs1	8 (6.84%)	3 (1.85%)	0.03472
Sulfonamides	sat2, sul1, sul2, sul3	19 (12.18%)	39 (18.06%)	0.12317
Tetracyclines	tet(A), tet(B)	15 (19.23%)	33 (30.56%)	0.08155
Multidrug efflux pump	acrF, emrD, mdtM, mdf(A)	0 (0%)	82 (37.96%)	2.88326E-18
Total		111 (5.81%)	372 (14.06%)	433319E−19
*Antimicrobial resistance associated mutations*				
Fluoroquinolones	gyrA_D87G, gyrA_D87N, gyrA_D87Y, gyrA_D87A, gyrA_S83L, parC_A56T, parC_E84G, parC_S80I, parC_S80R, parE_S458A, ptsI_V25I, uhpT_E350Q	13 (3.33%)	73 (13.52%)	4.42595E−08
Fosfomycin	cyaA_S352T	0 (0%)	9 (5.56%)	0.00387
Total		13 (2.56%)	82 (11.68%)	4.38744E−10

Among genes and mutations associated to resistance to critically important antimicrobial agents of the highest priority (HP-CIA) (WHO, 2019), 19 (1.1%) and 46 (1.9%) genes coding for extended spectrum beta-lactamases and associated to resistance to third generation cephalosporins (bla_TEM1,104,106,135_ bla_CTX-M-15_) were detected in the newly sequenced and public genomes respectively. Regarding resistance to fluoroquinolones, 9 (0.5%) and 7 (0.3%) genes and 13 (2.1%) and 75 (8.7%) mutations were detected respectively in newly sequenced and public genomes. No resistance genes or mutations were detected as associated to colistin resistance.

With a specific regard to multidrug resistance, newly sequenced genomes lack genes related to efflux pump transporters. When overexpressed, those genes are associated to multidrug resistance to a variety of antimicrobial classes (Li et al., 2015).

According to Chi Square test analysis, the lower incidence of antimicrobial resistance associated genes in the newly sequenced AB-free genomes of this study in comparison to the public genomes was statistically significant (*P* < 0.05) considering the whole group of genes as well as specifically for those genes conferring resistance to aminoglycosides, β-lactams, phenicols, trimethoprim, and lincosamides (Table 1). Significant reductions were assessed also in relation to the whole group of antimicrobial resistance associated mutations, as well as specifically for fluoroquinolones and fosfomycin resistance (Table 1).

Despite the strategies implemented for a more prudent use of antimicrobials in animal production for quite a few years, percentages of AMR bacteria in food animals continue to be high (EFSA and ECDC, 2021). This delay in observing an effect on AMR occurrence is most probably linked to the genetic bases of antimicrobial resistance. Genes conferring resistance to different antimicrobial classes are often located on the same mobile genetic element which can spread and persist among bacteria and the environment well after the antimicrobial agent was used or even in the absence of its employment (Malhotra-Kumar et al., 2016; Olaitan et al., 2016). The contribution of the food-producing environment as reservoir of AMR genes and bacteria was recently assessed by EFSA (Koutsoumanis et al., 2021). In this report, AMR genes and bacteria of the highest priority were particularly found in feces/manure, water and soil suggesting the importance to focus on: (1) reducing fecal microbial contamination and (2) minimizing persistence/recycling of AMR genes and bacteria in the animal production environment.

Although the 3° generation cephalosporin resistance associated genes found in this study represent a concern, a first indication of the impact of a more prudent use of antimicrobials on AMR is suggested. In particular, a lower occurrence of mutations associated to fluoroquinolone resistance was observed along with an overall lower number of AMR genes in *E. coli* genomes isolated from broiler carcasses produced without the use of antibiotics in Italy in 2019 in comparison to *E. coli* genomes isolated in previous years within the broiler production chain. More studies are needed in next years on a higher number of genomes to confirm this first observation.
